# Otologic complications of temporomandibular joint arthrocentesis due to arteriovenous fistula

**DOI:** 10.1002/ccr3.6235

**Published:** 2022-08-22

**Authors:** Joseph A. Fanti, Abhita Reddy, Evan Greenbaum, Mohammed Qaisi, Biraj Shah, James Murphy

**Affiliations:** ^1^ Department of Oral and Maxillofacial Surgery John H. Stroger Hospital Chicago Illinois United States; ^2^ Department of Otolaryngology Northwestern McGaw Medical Center Chicago Illinois United States

**Keywords:** arteriovenous fistula, embolization, hearing loss, superficial temporal artery, TMJ arthrocentesis

## Abstract

Arteriovenous fistula (AVF) is a rare sequela following arthrocentesis of the temporomandibular joint. This case report discusses a constellation of symptoms, findings, and appropriate management of a patient with a superficial temporal AVF. Several findings in this case, including vertigo, nystagmus, and hearing loss, have not been previously documented in the literature.

## Graphical abstract

Hearing loss as a result of an Arteriovenous fistula (AVF) is a rare sequela following temporomandibular joint arthrocentesis but can be readily managed with prompt diagnosis and embolization of the AVF.

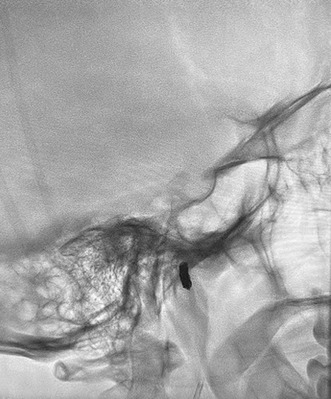

## 1 INTRODUCTION

Temporomandibular Joint (TMJ) arthrocentesis is a minimally invasive procedure that aimed to relieve pain symptoms and improve function in patients with an arthrogenous TMJ disorder. The procedure involves lavage of the TMJ, instillation of a steroidal medication and is typically performed without direct visualization of the joint. There are well‐documented complications associated with the procedure including extravasation of fluid into surrounding tissue, facial nerve injury (0.7%–0.6%), fifth nerve deficit (0.1%–2.4%), otic injury (0.5%–8.6%), preauricular hematoma, superficial temporal artery aneurysm, transarticular perforation, intracranial perforation, extradural hematoma, parapharyngeal swelling, and intra‐articular problems.^1^

Arteriovenous fistula is an aberrant communication between an artery and a vein where the capillary vasculature is bypassed. The etiologies of an AVF are trauma, iatrogenic, and congenital. Two theories aimed to elucidate the pathophysiology of a traumatic and iatrogenic AVF. The first mechanism involves simultaneous disruption of the artery and the adjacent vein with subsequent bridging of the artery and vein. The second mechanism begins with disruption of the vasa vasorum in the arterial wall. Proliferation of endothelial cells from the damaged vasa vasorum then forms numerous small vessels, leading to vascular communication channels between the artery and vein.^2^ The term Arteriovenous Malformation (AVM) is used to define a congenital AVF that is present at birth, or discovered shortly after, and grows commensurately with the child.^3^

## 2 CASE REPORT

**FIGURE 1:**
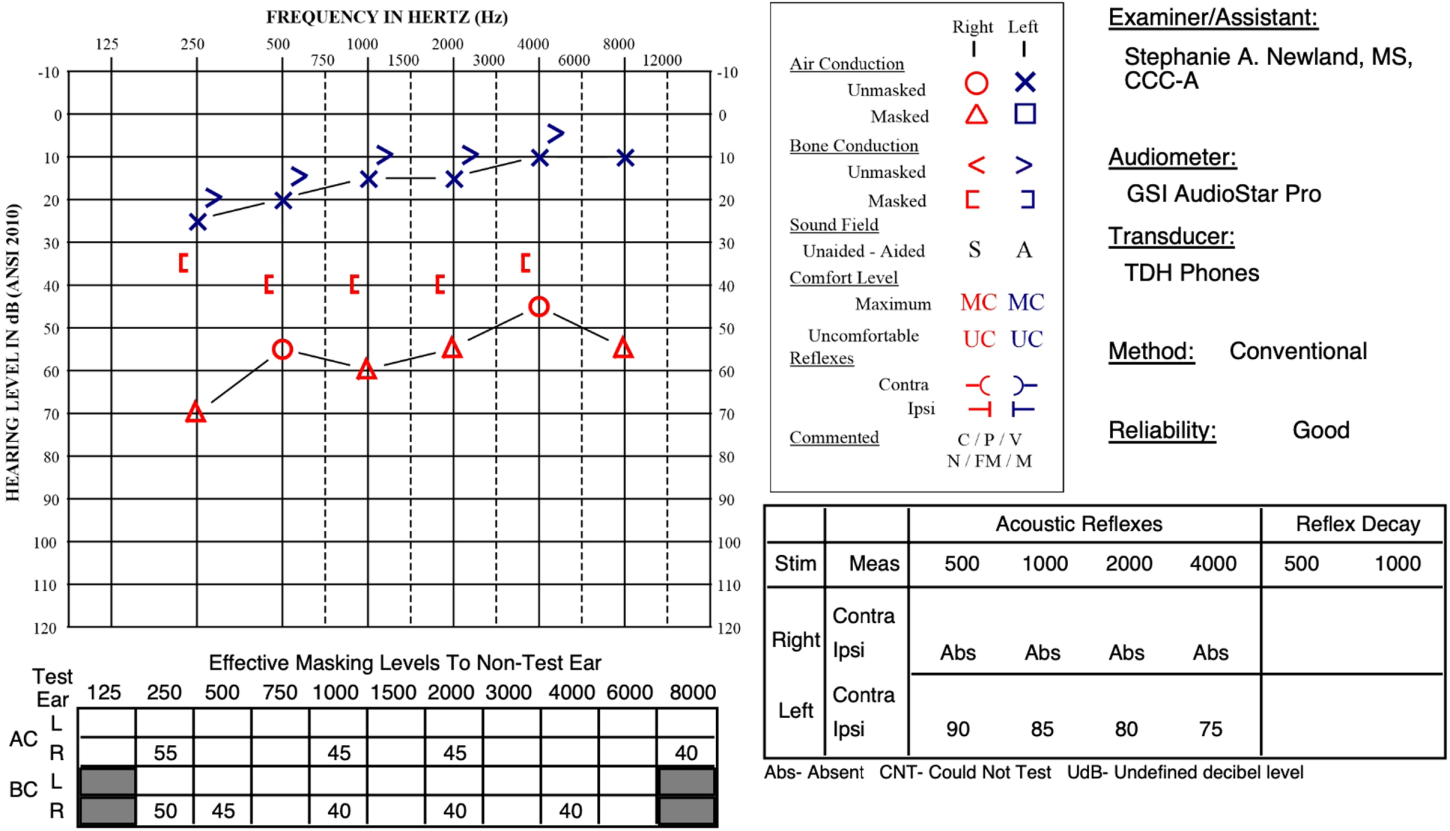
Initial audiogram postoperatively demonstrating moderate–severe to moderate mixed hearing loss. Also demonstrated is an absent right‐sided ipsilateral acoustic reflex, while word recognition is preserved

**FIGURE 2:**
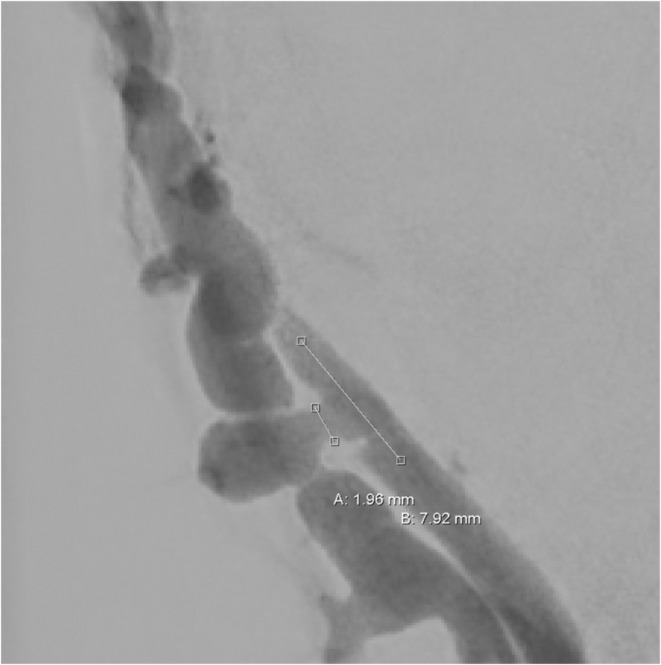
Diagnostic angiogram demonstrates the Arteriovenous fistula (AVF) of the superficial temporal artery and vein. Point A shows a bridging vessel of 1.96 mm between the artery on the right and the vein on the left. Point B is the planned region of embolization within the artery

**FIGURE 3:**
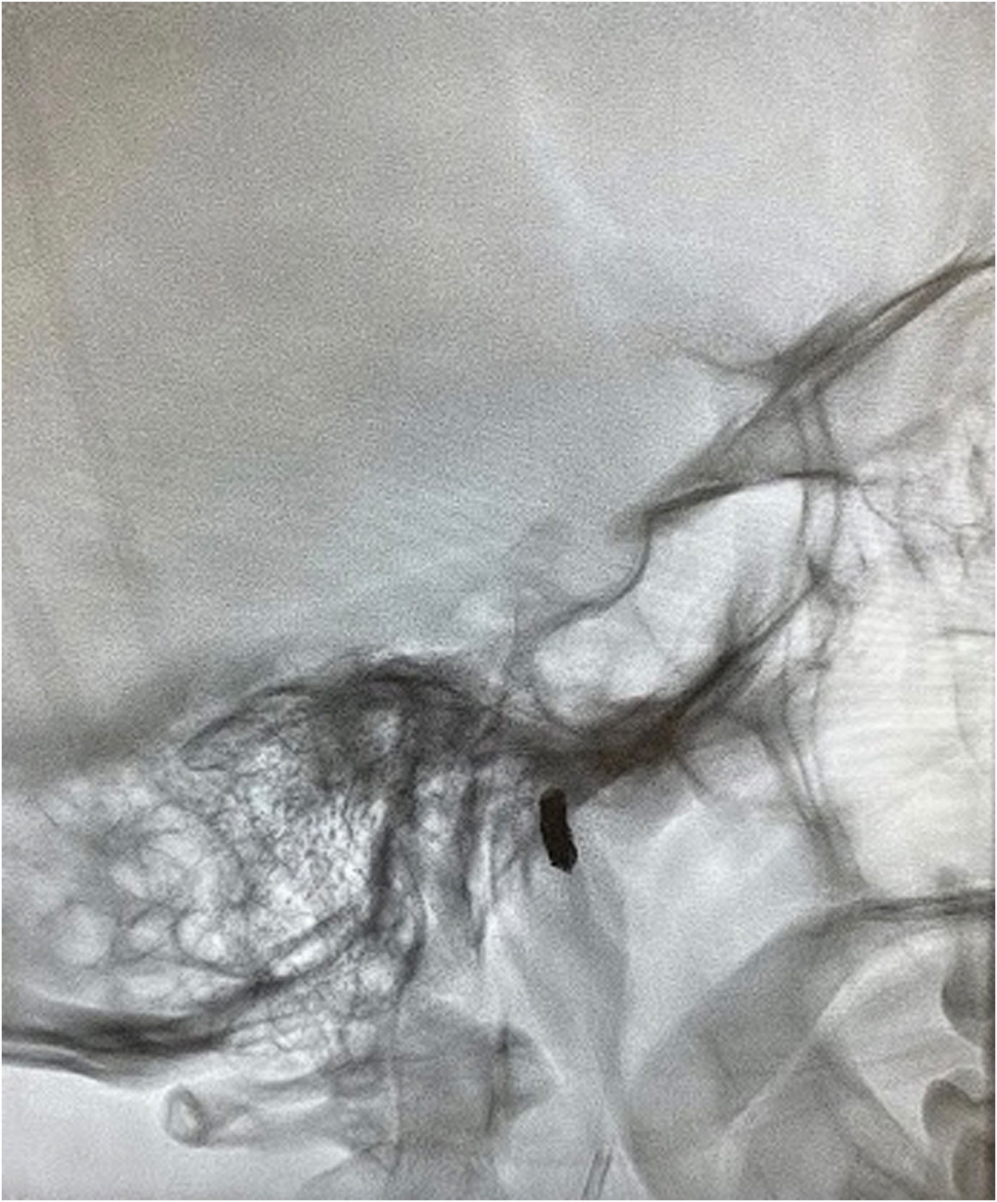
Plain film radiograph demonstrates embolization of the superficial temporal artery with coils

**FIGURE 4:**
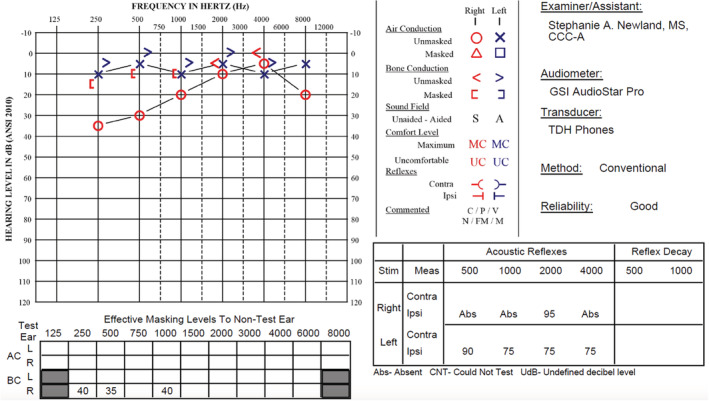
Postembolization audiogram demonstrating right‐sided mild conductive hearing loss in low frequencies

A 37‐year‐old woman with no significant past medical history presented with bilateral 8/10 pain localized to the preauricular region, and with positive direct and indirect Mahan's sign. Bilateral diagnostic lidocaine blocks diminished pain symptoms to 2/10, thus the decision was made to take the patient to the operating room for bilateral arthrocentesis. The procedure was carried out in standard fashion where the inflow and outflow ports were inserted into their respective points based on the Holmlund‐Hellsing line. The joint space was irrigated with normal saline and injected with Kenalog. Upon removal of the right TMJ inflow port, small pulsatile bleeding was noted and controlled with gauze pressure for 2 min. One week postoperatively, the patient complained of loud right‐sided constant pulsatile tinnitus, diminished right‐sided hearing, and continued pain on the right side. The left TMJ pain had diminished to 2/10. Symptoms on the right persisted, and on postoperative week three, the patient developed a palpable preauricular thrill, constant debilitating vertigo, and bilateral right‐beating horizontal nystagmus. Patient could not tolerate Weber and Rinne testing. An audiogram revealed right‐sided moderate–severe mixed hearing loss in all frequencies, which was improved by applying manual pressure over the palpable thrill (Figure 1). A CT angiogram and subsequent diagnostic angiogram revealed an AVF of the superficial temporal vessels (Figure 2). The AVF was embolized and coiled by neuroradiology under conscious sedation (Figure 3). The patient reported immediate resolution of pulsatile tinnitus, vertigo, and hearing loss at the conclusion of her procedure, confirmed by repeat audiogram (Figure 4).

## 3 DISCUSSION

Anatomy surrounding the TMJ is susceptible to disruption, which can lead to complications following arthrocentesis. The superficial temporal artery arises as a direct branch from the external carotid artery. It courses along the posterior margin of the mandibular condyle at the level of temporoparietal fascia and proceeds to course over the posterior root of the zygomatic arch. Above the zygomatic arch, it divides into the frontal and parietal branches.^4^ The superficial temporal vein is formed by the union of the parietal and frontal veins, which drain the pterygoid venous plexus. The superficial temporal vein then descends into the parotid gland and joins the transverse facial vein to become the retromandibular vein.^5^ The anatomic location of the vessels is within the region where inflow and outflow port needles are inserted during arthrocentesis.

Blood supply to the middle and inner ear includes anastomoses with the superficial temporal artery via the posterior auricular artery. Many of the patient's findings may be due to disruption in vasculature to her middle and inner ear, as evidenced by mixed hearing loss on initial audiogram. Additionally, the stapedial artery originates from the stylomastoid branch of the posterior auricular artery and provides blood supply to the stapedius muscle. While the presented audiologic evaluation does not include complete acoustic reflex testing, it is possible the absence of a right‐sided ipsilateral acoustic reflex was due to disruption of blood supply to the stapedius muscle.

## 4 CONCLUSION

Complications from TMJ arthrocentesis may include otologic findings, which the otolaryngologist should be apprised of. Currently, no documentation exists in the literature to the authors' knowledge regarding traumatic superficial temporal arteriovenous fistula causing vertigo, nystagmus, and hearing loss after TMJ arthrocentesis.

## AUTHOR CONTRIBUTIONS

Joseph Fanti and Abitha Reddy were both involved in the patient care and cowrote the paper. Drs. Greenbaum, Qaisi, Shah, and Murphy were all integral in patient management, audiogram testing, editing, and theorizing.

## CONSENT

Written informed consent was obtained from the patient to publish this report in accordance with the journal's patient consent policy.

## Data Availability

Due to privacy and ethical concerns, neither the data nor the source of the data can be made available.
